# MiR‐4524b‐5p‐targeting ALDH1A3 attenuates the proliferation and radioresistance of glioblastoma via PI3K/AKT/mTOR signaling

**DOI:** 10.1111/cns.14396

**Published:** 2023-08-08

**Authors:** Hai Yu, Xiaodong Li, Yi Li, Tuo Wang, Maode Wang, Ping Mao

**Affiliations:** ^1^ Department of Neurosurgery The First Affiliated Hospital of Xi'an Jiaotong University Xi'an Shaanxi China; ^2^ Center of Brain Science The First Affiliated Hospital of Xi'an Jiaotong University Xi'an Shaanxi China; ^3^ Department of Radiotherapy The First Affiliated Hospital of Xi'an Jiaotong University Xi'an Shaanxi China

**Keywords:** ALDH1A3, glioblastoma, miR‐4524b‐5p, PI3K/AKT/mTOR signaling, radioresistance, tumor proliferation

## Abstract

Increasing evidence has revealed a strong connection between the aldehyde dehydrogenase family member ALDH1A3 and tumorigenesis, therapy resistance, and prognosis in diverse types of cancer. However, the specific miRNA involved in the pathways that regulate ALDH1A3‐mediated glioblastoma (GBM) radioresistance remains to be elucidated. In this study, we demonstrated a high expression of ALDH1A3 in GBM cells, which plays a critical role in their proliferation and radioresistance. We also identified miR‐4524b‐5p, which is downregulated in GBM, as the ALDH1A3 upstream regulator. Overexpression of miR‐4524b‐5p reduced proliferation and radioresistance in GBM cells. Moreover, silencing ALDH1A3 reduced PI3K/AKT/mTOR signaling and glycolytic activity in GBM cells, whereas inhibiting mTOR reversed the radioresistance effects of ALDH1A3 on these cells. In vivo experiments have evidenced that ALDH1A3 silencing and miR‐4524b‐5p overexpression significantly reduced tumor growth and GBM cells radioresistance. In summary, targeting the miR‐4524b‐5p and ALDH1A3 axis is a promising therapeutic strategy for treating GBM.

## Graphical abstract

MiR‐4524b‐5p targets ALDH1A3 to attenuate proliferation and radioresistance in GBM via the regulation of PI3K/AKT/mTOR signaling pathways and glycolysis activity.

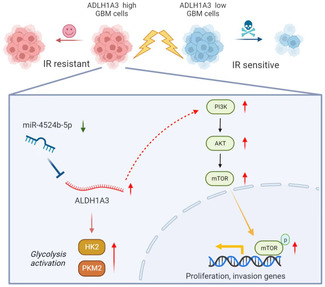

## 1 INTRODUCTION

Glioblastoma (GBM) is one of the most common and fatal primary tumors of the central nervous system in adults.^1^ Current approaches for GBM treatment involve a combination of maximum surgical resection and radiotherapy plus concomitant and adjuvant temozolomide. However, the overall survival rate for GBM patients is low.^2^ It has been demonstrated that the development of radioresistance leads to tumor recurrence, significantly impairing the prognosis of GBM patients.^3^ In addition, increasing evidence has revealed a strong connection between ALDH1A3 and radioresistance in distinct types of cancer.^4^, ^5^, ^6^ Therefore, it is important to investigate the role and mechanism of ALDH1A3 in radioresistance to identify a novel therapeutic target for GBM treatment.

The human ALDH superfamily comprises 19 active ALDH genes that play an essential role in metabolic processes by oxidizing aldehydes into carboxylic acids.^7^ Furthermore, ALDH1A3, a significant member of the ALDH family, has been associated with tumorigenesis, progression, and radioresistance in various types of cancer.^8^ Recent studies have shown that miR‐7 targets ALDH1A3, which leads to a reduction of breast cancer growth and stem cell subpopulations.^9^ Inhibition of ALDH1A3 results in a decrease in invasion and metastasis in colorectal cancer cells through the miR‐200‐ZEB1/SANI2 pathway.^10^ However, the role of upstream miRNA in regulating radioresistance through ALDH1A3 signaling pathways in GBM remains to be elucidated.

MicroRNAs (miRNAs) are small, endogenous, non‐coding nucleotides that bind to mRNA strands to degrade or prevent them from being translated into proteins.^11^ Aberrant expression of miRNAs has been reported to promote tumorigenesis and increase resistance to radiation in a variety of cancers.^12^, ^13^, ^14^ A recent study has revealed that overexpressing miR‐195‐3p inhibits nasopharyngeal carcinoma growth, progression, and radioresistance by suppressing cyclin‐dependent kinase 1.^15^ Moreover, miR‐153‐3p enhances cell radiosensitivity by targeting BCL2.^16^ It has been also reported that miR‐187 promotes the proliferation of prostate cancer cells by inhibiting the expression of ALDH1A3.^17^ However, the potential of miRNA to interact with ALDH1A3 in the regulation of tumorigenesis and radioresistance in GBM remains to be investigated.

In the present study, we showed that increased expression of ALDH1A3 promotes tumorigenesis and radioresistance in GBM. Moreover, we have demonstrated that miR‐4524b‐5p targets ALDH1A3 to reduce proliferation and radioresistance in GBM by regulating the PI3K/AKT/mTOR signaling pathway and glycolytic activity. Therefore, targeting the miR‐4524b‐5p‐ALDH1A3 axis may be a promising therapeutic approach for treating GBM.

## 2 MATERIALS AND METHODS

### 2.1 Cell culture

The GBM cell lines U251, A172, U87, and LN229, and normal human astrocytes (NHA) were obtained from the Cell Bank of Type Culture Collection of the Chinese Academy of Sciences. Cells were cultured in DMEM (Gibco), containing 10% fetal bovine serum (FBS; Gibco) and 1% penicillin–streptomycin solution (Thermo Fisher Scientific, Inc.) and maintained in a humidified incubator at 37°C with 5% CO_2_ and 95% air.

### 2.2 Clinical samples

We obtained 13 pairs of GBM and adjacent normal tissues from glioma patients, undergoing brain surgery at The First Affiliated Hospital of Xi'an Jiaotong University, between June and July 2021. The use of these tissues was approved by the Hospital's Ethics Committee (Xi'an, Shaanxi, China 710,061; Approval No. 2020‐G13) and consent forms were signed for the use of clinical samples.

### 2.3 X‐ray irradiation

U251 GBM cells and BALB/c nude female mice were exposed to X‐ray irradiation at 0, 2, 4, 6, and 8 Gy for 0, 24, 48, and 72 h, using a small animal radiation research platform (SARRP; Xstrahl Ltd.). Mice were anesthetized using a portable machine (VetTech Solutions Ltd), before being placed in the irradiation device. Following irradiation, they were kept in cages and monitored, until they regained full consciousness.

### 2.4 Cell transfection

miR‐4524b‐5p mimics (5′‐AUAGCAGCAUAAGCCUGUCUC‐3′), mimic negative control (mimic NC; cat. No. miR1N0000001–1‐10), miR‐4524b‐5p inhibitor (5′‐GAGACAGGCUUAUGCUGCUAU‐3′), and a negative control inhibitor (cat. No. miR2N0000001‐1‐10) were purchased from RIBOBIO Co., Ltd. GenePharma provided pcDNA3.1 and pcDNA3.1‐ALDH1A3 plasmids. U251 cells were cultured in a six‐well plate and incubated overnight at 37°C with 70% confluence. We used the Lipofectamine® 3000 kit (Thermo Fisher Scientific, Inc.), following the manufacturer's instructions to transfect plasmids into cells. Cells were then collected 48 h after transfection for further analysis. Transfection efficiency was evaluated by qRT‐PCR and Western blot.

### 2.5 RNA isolation and quantitative PCR

We used the TRIzol® reagent (AccuRef Scientific) to extract RNA, following the supplier's instructions. RNA concentration was measured in a NanoDrop 2000 device (Thermo Fisher Scientific, Inc.) and cDNA was synthesized with the PrimeScript® RT Master Mix Perfect Real‐Time Reagent kit (Takara Biotechnology Co., Ltd.). Furthermore, qRT‐PCR was performed on an ABI 7500 Real‐Time PCR instrument (Applied Biosystems), using cDNA and SYBR Green Reagent (Takara Biotechnology Co., Ltd.), under the following conditions: 95°C for 5 min, then 40 cycles of 95°C for 15 s, 58°C for 20 s, and 72°C for 10 s. The internal control was either GAPDH (mRNA) or U6 (miRNA). Relative quantification was calculated as 2^−ΔCT^. ΔCt values were calculated by subtracting the mean Ct value of the control gene from the mean Ct value of the target gene. The following primers were used in this study: ALDH1A3 forward, GATAAGCCCGACGTGGACAA and reverse, ATACAGCCCTCCAGGTCGAT; miR‐4524b‐5p forward, CGCGATAGCAGCATAAGCC and reverse, AGTGCAGGGTCCGAGGTATT; HK2 forward, GTGAATCGGAGAGGTCCCAC and reverse, CAAGCAGATGCGAGGCAATC; PKM2 forward, GCCGAGAGCCAAGAAAAGAC and reverse, GCCCTTTCGGTGGGACTAAA; GAPDH forward, GGAGCGAGATCCCTCCAAAAT and reverse GGCTGTTGTCATACTTCTCATGG; U6 forward, CTCGCTTCGGCAGCACA and reverse AACGCTTCACGAATTTGCGT.

### 2.6 Western blot

We used the RIPA lysis buffer (AccuRef Scientific) to extract total proteins from cells that were quantified by the BCA method (Thermo Fisher Scientific, Inc.). An equal volume of loading buffer was added to the protein before subjecting it to 12% SDS‐PAGE separation. The separated protein was then electronically transferred to a PVDF membrane (MilliporeSigma), which was blocked with 5% skim milk for 1 h at room temperature. Next, the membrane was incubated overnight at 4°C with primary antibodies, including anti‐ALDH1A3 (Abcam; ab129815), anti‐PI3K (Abcam; ab140307), anti‐pAKT‐Ser473 (Proteintech; 80455‐1‐RR), anti‐AKT (Proteintech; 60203‐2‐Ig), anti‐pmTOR‐Ser2448 (Proteintech; 67778‐1‐Ig), anti‐mTOR‐Ser2448 (Proteintech; 66,888‐1‐Ig), anti‐HK2 (Proteintech; 66974‐1‐Ig), anti‐PKM (Proteintech; 10,078‐2‐AP), and anti‐β‐actin (Proteintech; 81,115‐1‐RR). After the initial incubation, membranes were exposed to a secondary antibody conjugated with HRP for 1 h at room temperature. Protein bands were visualized using the Enhanced Chemiluminescence kit (AccuRef Scientific) and analyzed using the ImageJ software (version 1.49; National Institutes of Health).

### 2.7 Cell counting kit‐8 assay

We used the CCK‐8 kit (AccuRef Scientific) to assess cell proliferation. U251 cells (2000 cells/well) were cultured in 96‐well plates with different treatments. Ten microliters of the CCK‐8 reagent were added to all wells and plates incubated for 2 h at 37°C. Absorbances were then read at 450 nm in an ELX800 microplate reader (BioTek Instruments, Inc.) to evaluate cell proliferation.

### 2.8 Colony forming assay

U251 cells were incubated in 6‐well plates at a density of 500 or 1000 cells per well for 14 days, refreshing the culture medium every other day. Next, cell colonies were fixed with 100% methanol at 4°C for 30 min and stained with 1% crystal violet in 20% methanol at room temperature for 30 min. Cell clones were observed using an inverted microscope.

### 2.9 Flow cytometry

The Annexin V FITC Apoptosis Detection kit was purchased from AccuRef Scientific and apoptosis analysis was performed following the manufacturer's instructions. The level of apoptosis was determined by calculating the percentage of cells in the early and late stages of apoptosis.

### 2.10 Dual‐luciferase reporter assay

The luciferase reporter gene vectors PGL3 and PGL3‐ALDH1A3, containing the ALDH1A3 promoter, were purchased from Genepharm. The plasmid containing the reporter gene was co‐transfected with phRL‐TK into cells that were cultured to a confluence of 70% to 80% in 24‐well plates. U251 cells were co‐transfected with 100 ng of pGL3‐WT, 20 ng of the transfection control Renilla vector (pRL‐TK; Promega Corporation), and 100 nM of miR‐4524b‐5p mimic or negative control (NC) mimic (RIBOBIO Co., Ltd.), using the Lipofectamine® 3000 kit (Thermo Fisher Scientific, Inc.) and following supplier's instructions.

### 2.11 Measurement of glucose uptake and lactate and ATP production

The impact of silencing ALDH1A3 on glucose uptake and lactate and ATP production in U251 cells was measured using the colorimetric glucose uptake assay kit (Abcam; cat. no. ab136955), the colorimetric lactate assay kit (Abcam; cat. No. ab65331), and the luminescent ATP detection assay kit (Abcam; cat. No. ab113849), following manufacturer's protocols.

### 2.12 Xenograft mouse model

Female BALB/c nude mice, aged 4–6 weeks, were purchased from Jiangsu Aniphe Biolaboratory Inc. They were housed in a specific‐pathogen‐free (SPF) environment at the Experimental Animal Center of The First Affiliated Hospital of Xi'an Jiaotong University. Stable negative control lentivirus‐infected U251 cells (LV) and stable lentivirus‐infected U251 cells expressing miR‐4524b‐5p (LV‐miR‐4524b‐5p) were suspended in PBS and mixed with an isopycnic Matrigel (BD Biosciences) at a final concentration of 5 × 10^6^ cells/mL. Next, 1 × 10^6^ cells were subcutaneously injected into nude mice, and tumor size was measured every 3 days, using Vernier calipers. Mice were then euthanized with an intraperitoneal injection of 180 mg/kg ketamine (Sigma‐Aldrich) and 20 mg/kg xylazine (Sigma‐Aldrich) at day 27, after which subcutaneous tumors were weighed, imaged, and analyzed. All animal experiments were conducted following the guidelines outlined in the “Guide for the Care and Use of Laboratory Animals” (National Academies Press). The animal experimental protocol was approved by the Ethical Review Committee for Animal Experiments of the First Affiliated Hospital of Xi'an Jiaotong University.

### 2.13 Immunohistochemistry staining

Mouse tumor slices were incubated overnight with primary antibodies (1:200 dilution) at 4°C. After washing with PBS, slices were incubated with HRP‐conjugated secondary antibodies (1:1000 dilution) for 1 h at room temperature. Immune signals were then detected using the DAB substrate kit (Vector Laboratories) and nuclei were counterstained with hematoxylin and Hoechst. Negative controls consisted of slices that were not exposed to primary antibodies. The following antibodies were used in this study: anti‐Ki67 primary antibody (Proteintech; 27,309‐1‐AP; rabbit), anti‐pmTOR primary antibody (Proteintech; 67,778‐1‐Ig; mouse), IgG (Proteintech; 30,000‐0‐AP; rabbit; negative control), goat anti‐rabbit secondary antibody (Abcam; ab150077; goat; horseradish peroxidase‐conjugated), and goat anti‐mouse secondary antibody (Abcam; ab150113; goat; horseradish peroxidase‐conjugated).

### 2.14 TUNEL assay

Apoptosis detection was performed using the in situ cell death detection kit POD (Roche Diagnostics). The procedure involved fixing coverslips with 4% paraformaldehyde, blocking with 3% H_2_O_2_, and washing twice with PBS. Coverslips were then permeated with 0.1% Triton X‐100 on ice for 2 min, after which they were exposed to the TUNEL reagent in darkness at 37°C for 1 h in a humid chamber. After three washes, slides were observed by fluorescence microscopy (Olympus).

### 2.15 Bioinformatics analysis

Overall survival rates for ALDH1A3 and miR‐4524b‐5p in different grades of glioma, according to the World Health Organization (WHO), were obtained and analyzed using data from the Chinese Glioma Genome Atlas (CGGA) database (http://www.cgga.org.cn/). The ENCOR1 (https://starbase.sysu.edu.cn/) and TargetScan (https://www.targetscan.org) databases were utilized to identify potential miRNAs targeting ALDH1A3.

### 2.16 Statistical analysis

Statistical analysis was determined using the SPSS 22.0 software (IBM). Data represented the mean ± standard deviation of triplicate determinations from three independent experiments. The normality of data distribution was assessed using the Shapiro–Wilk test. We used the two‐tailed Student *t*‐test (normal distribution) or the Mann–Whitney test (nonnormal distribution) for the comparison between the two groups. Comparisons among multiple groups were estimated by one‐way or two‐way ANOVA, followed by Bonferroni's post hoc multiple comparison tests (normal distribution) or the Kruskal‐Wallis's test (nonnormal distribution).

## 3 RESULTS

### 3.1 Increased expression of ALDH1A3 promotes proliferation and radioresistance of GBM cells

**FIGURE 1:**
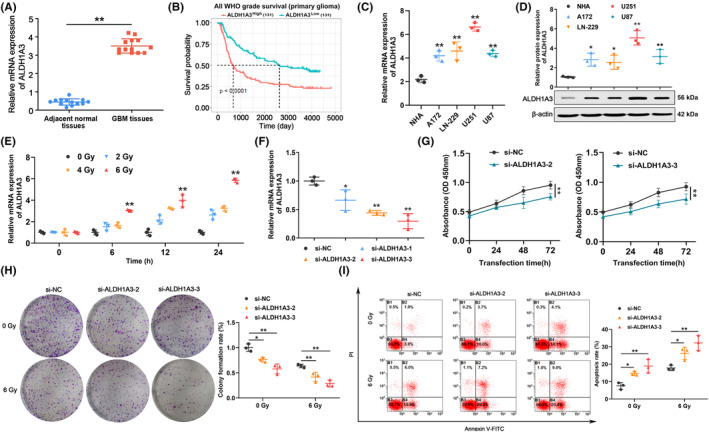
Increased expression of ALDH1A3 promotes proliferation and radioresistance in GBM cells. (A) ALDH1A3 was highly expressed in GBM tissues, compared with adjacent normal tissues. (B) The Kaplan–Meier analysis of the CGGA database revealed that glioma patients exhibiting elevated levels of ALDH1A3 expression had a poor clinical prognosis. (C) mRNA expression levels of ALDH1A3 were elevated in GBM cell lines, compared with that of normal human astrocytes (NHA). (D) The amount of ALDH1A3 protein was enriched in GBM cell lines, compared with that of NHA. (E) mRNA expression levels of ALDH1A3 were measured in U251 cells exposed to 0, 2, 4, and 6 Gy doses of radiation for 0, 6, 12, and 24 h. (F) Levels of ALDH1A3 mRNA decreased in U251 cells, after siRNA transfection. (G) Silencing ALDH1A reduced U251 cells growth rate. (H) U251 cells colony‐forming potential was significantly reduced, after transfection with si‐ALDH1A3–2 and si‐ALDH1A3–3 and upon exposure to 0 Gy and 6 Gy radiation. (I) Silencing ALDH1A3 and exposing GBM cells to 6 Gy radiation significantly increased U251 cells apoptosis rates. NC represents the negative control. β‐actin served as a control. *p < 0.05, **p < 0.01.

Thirteen sets of GBM tissues were examined to determine the expression of ALDH1A3, using qRT‐PCR. Results showed that ALDH1A3 expression levels were significantly higher in GBM tissues, compared with adjacent normal tissues (Figure 1A). Bioinformatics analysis of the CGGA database revealed that glioma patients exhibiting high levels of ALDH1A3 expression had a poor clinical prognosis (Figure 1B). Moreover, qRT‐PCR and Western blot analyses were conducted to evaluate mRNA and protein expression levels of ALDH1A3. As shown in Figure 1C,D, U251 cells exhibited higher expression of ALDH1A3, compared with normal human astrocytes (NHA) and other GBM cell lines. ALDH1A3 has been reported to play a role in chemoresistance in several types of cancer.^18^, ^19^, ^20^, ^21^ However, the role of ALDH1A3 on GBM radioresistance remains to be elucidated. To explore the potential correlation between ALDH1A3 and radioresistance, we assessed the mRNA expression of ALDH1A3 at different radiation doses and exposure times. As shown in Figure 1E, ALDH1A3 expression increased in U251 cells, which correlated with increased exposure to radiation doses and duration. Furthermore, three siRNAs were designed to silence the expression of ALDH1A3 in GBM cells. Among them, si‐ALDH1A3‐2 and si‐ALDH1A3‐3 were found to be the most effective in targeting ALDH1A3 (Figure 1F). Growth and colony‐forming potential of U251 cells were significantly reduced after transfection with si‐ALDH1A3‐2 and si‐ALDH1A3‐3, compared with the si‐negative control (si‐NC) (Figure 1G). As shown in Figure 1H, si‐NC‐transfected U251 cells had a higher colony formation rate than cells treated with si‐ALDH1A3–2 or si‐ALDH1A3–3, after radiation exposure. Our results also indicated that silencing ALDH1A3 significantly increased U251 cells apoptosis rates, particularly those exposed to 6 Gy radiation, thus suggesting that suppressing ALDH1A3 may enhance the radiosensitivity of GBM cell lines (Figure 1I).

### 3.2 Decreased expression of miR‐4524b‐5p in GBM cells

**FIGURE 2:**
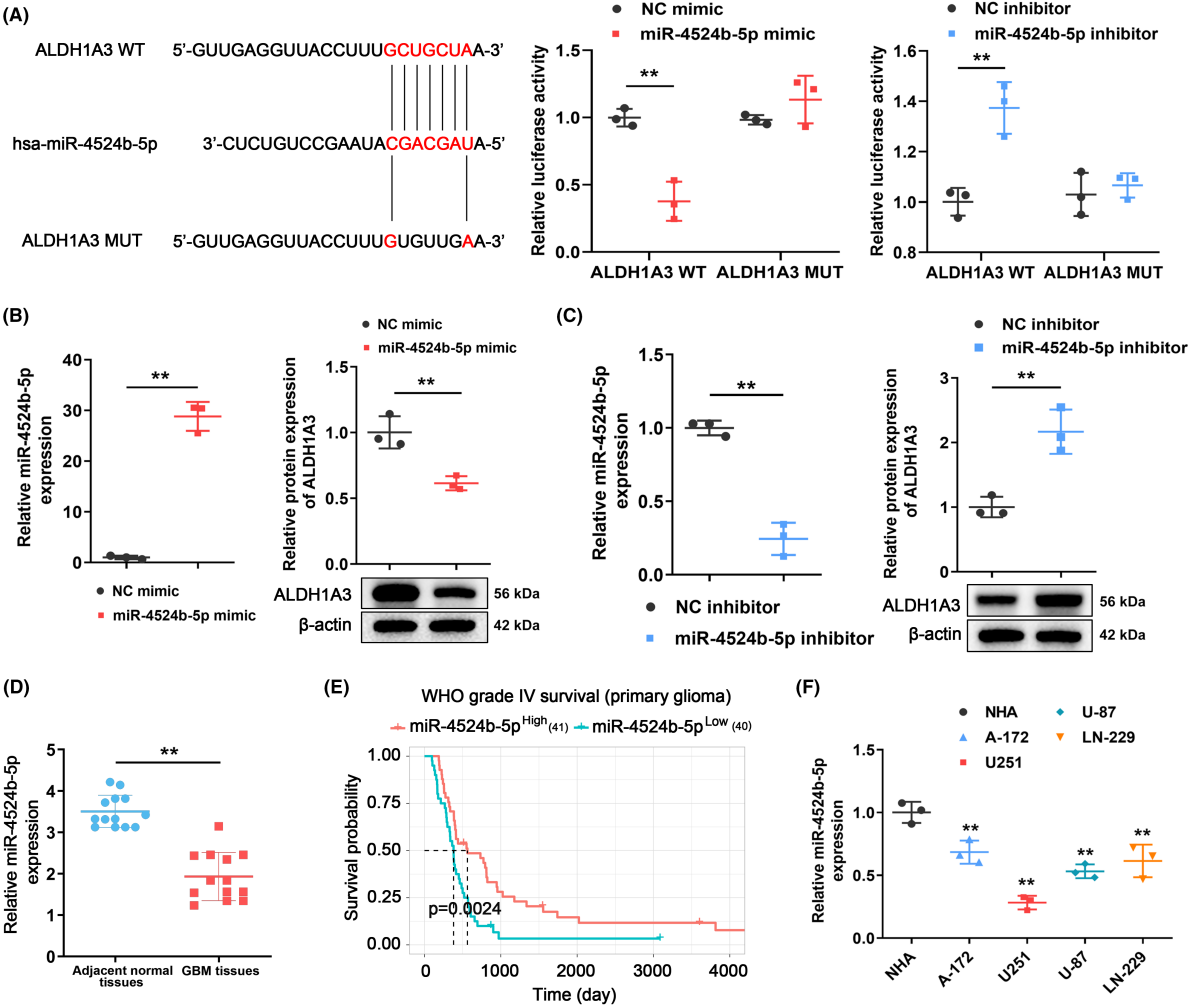
Decreased expression of miR‐4524b‐5p in GBM cells. (A) Luciferase activity significantly decreased, after co‐transfecting miR‐4524b‐5p mimics and ALDH1A3 WT. In contrast, luciferase activity increased in U251 cells that were co‐transfected with si‐miR‐4524b‐5p and ALDH1A3 WT. (B) Overexpression of miR‐4524b‐5p inhibited ALDH1A3 expression in U251 cells. (C) Inhibition of miR‐4524b‐5p increased ALDH1A3 expression in U251 cells. (D) Adjacent normal tissues exhibited higher expression of miR‐4524b‐5p, compared with GBM tissues. (E) The Kaplan–Meier analysis of the CGGA database revealed that GBM patients with higher levels of miR‐4524b‐5p experienced longer survival times. (F) GBM cell lines exhibited a decrease in the expression of miR‐4524b‐5p in comparison with normal human astrocytes (NHA). NC represents the negative control. **p < 0.01.

Bioinformatics analysis was conducted to identify the miRNA that binds to the ALDH1A3 consensus binding sites. As a result, miR‐4524b‐5p was identified as a potential miRNA that regulates the expression of ALDH1A3. To confirm the impact of miR‐4524b‐5p on ALDH1A3, a mutated version of ALDH1A3 was constructed (Figure 2A). Luciferase activities were significantly reduced in U251 cells that were co‐transfected with the miR‐4524b‐5p mimic and ALDH1A3 wild type (WT), compared with the control. In contrast, an increase in luciferase activity was observed in U251 cells that were co‐transfected with si‐miR‐4524b‐5p and ALDH1A3 WT (Figure 2A). However, the ALDH1A3 mutant (MUT) evidenced no effect on luciferase activity in U251 cells, during co‐transfection with miR‐4524b‐5p (Figure 2A). Moreover, overactivation of miR‐4524b‐5p resulted in a reduction of ALDH1A3 expression in U251 cells (Figure 2B). In contrast, inhibition of miR‐4524b‐5p increased ALDH1A3 expression in U251 cells (Figure 2C). Adjacent normal tissues exhibited a higher level of miR‐4524b‐5p expression, compared with GBM tissues (Figure 2D). As shown in Figure 2E, GBM patients with higher levels of miR‐4524b‐5p had a longer survival rate based on the Kaplan–Meier analysis of the CGGA database. Furthermore, U251 cells exhibited the lowest level of miR‐4524b‐5p expression in comparison with other GBM cell lines (Figure 2F). Therefore, miR‐4524b‐5p was significantly decreased in GBM.

### 3.3 Overexpression of miR‐4524b‐5p reduced proliferation and enhanced radiosensitivity in GBM cells

**FIGURE 3:**
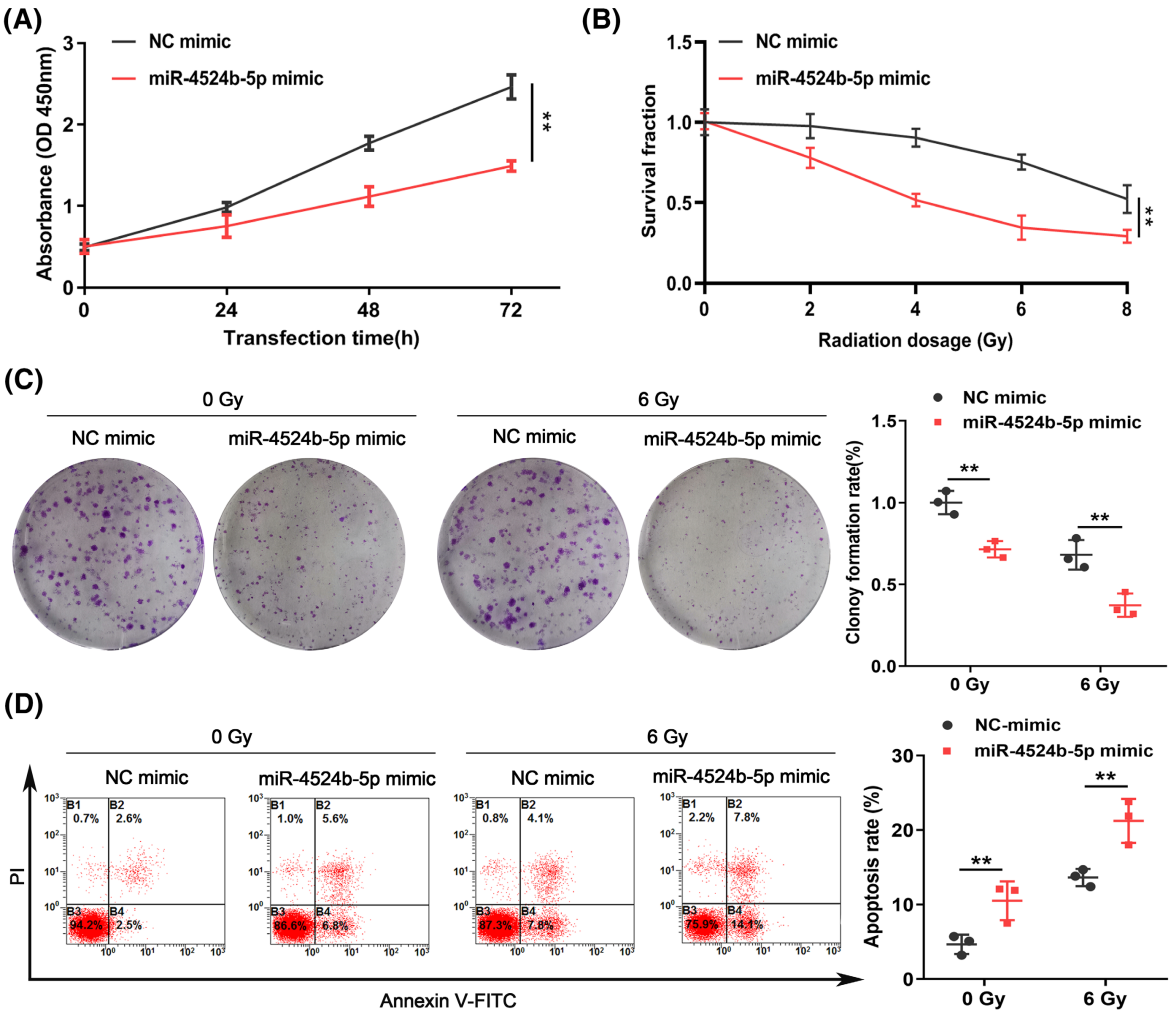
Overexpression of miR‐4524b‐5p reduced proliferation and enhanced radiosensitivity in GBM cells. (A) Overexpression of miR‐4524b‐5p reduced U251 cells proliferation. (B) U251 cells viability significantly decreased, after exposure to 2, 4, 6, and 8 Gy radiation levels, with miR‐4524b‐5p overexpression. (C) Overexpression of miR‐4524b‐5p significantly decreased U251 cells colony‐forming rate, particularly when exposed to 6 Gy radiation. (D) Overexpression of miR‐4524b‐5p and treatment with 6 Gy radiation significantly increased U251 cells apoptosis rates. NC represents the negative control. **p < 0.01.

As shown in Figure 3A, overexpression of miR‐4524b‐5p inhibited U251 cells proliferation. In addition, the sensitivity of U251 cells to radiation increased following transfection of miR‐4524b‐5p mimic after exposure to varying doses of radiation (Figure 3B). Furthermore, a significant decrease of U251 cells colony‐forming potential and overexpression of miR‐4524b‐5p were observed after exposure to 6 Gy radiation (Figure 3C). A significant increase in U251 cells apoptosis rates, following the combination of overexpression of miR‐4524b‐5p and 6 Gy radiation, indicates that miR‐4524b‐5p plays a crucial role in the response of GBM cells to radiation (Figure 3D). Taken together, overexpression of miR‐4524b‐5p not only inhibited GBM cells growth but also increased their radiosensitivity.

### 3.4 ALDH1A3 knockdown suppressed the activity of the PI3K/AKT/mTOR signaling pathway and glycolysis in GBM cells

**FIGURE 4:**
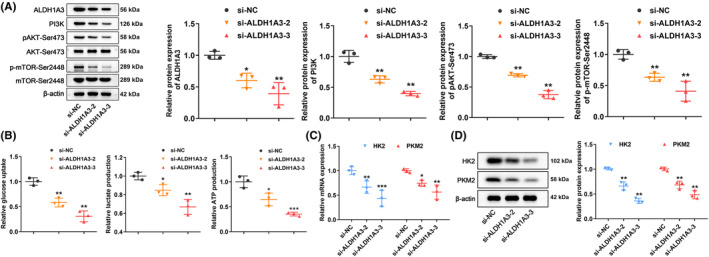
ALDH1A3 knockdown suppressed the activity of the PI3K/AKT/mTOR signaling pathway and glycolysis in GBM cells. (A) Activities of the PI3K/AKT/mTOR signaling pathways were significantly inhibited in U251 cells, after ALDH1A3 silencing. (B) Decrease glucose consumption, and lactate and ATP production in U251 cells, after ALDH1A3 silencing. (C) mRNA expression levels of HK2 and PKM2 were suppressed, following ALDH1A3 knockdown. (D) HK2 and PKM2 protein levels decreased, following ALDH1A3 inhibition. NC represents the negative control. *p < 0.05, **p < 0.01.

Recent studies have demonstrated that tumor radioresistance is linked to aberrant activation of the PI3K/AKT/mTOR pathway and glycolysis.^22^, ^23^ We performed multiple Western blot tests to investigate the impact of ALDH1A3 on the regulation of the PI3K/AKT/mTOR pathway and glycolysis. As demonstrated in Figure 4A, the activities in PI3K/AKT/mTOR signaling pathways were significantly inhibited in U251 cells, when ALDH1A3 was silenced. Furthermore, a reduction in glucose consumption, and lactate and ATP production were observed in U251 cells, after silencing ALDH1A3 (Figure 4B). HK2 and PKM2, the key enzymes of glycolysis, were examined by qRT‐PCR and Western blot, after ALDH1A3 knockdown in U251 cells. Results showed that ALDH1A3 decrease was associated with a reduction of mRNA and protein levels of HK2 and PKM2 in U251 cells (Figure 4C,D). These results indicated that suppression of ALDH1A3 impaired GBM cells activity in the PI3K/AKT/mTOR signal pathway and glycolysis.

### 3.5 Inhibition of mTOR enhanced radiosensitivity and counteracted the effects of ALDH1A3 on radioresistance

**FIGURE 5:**
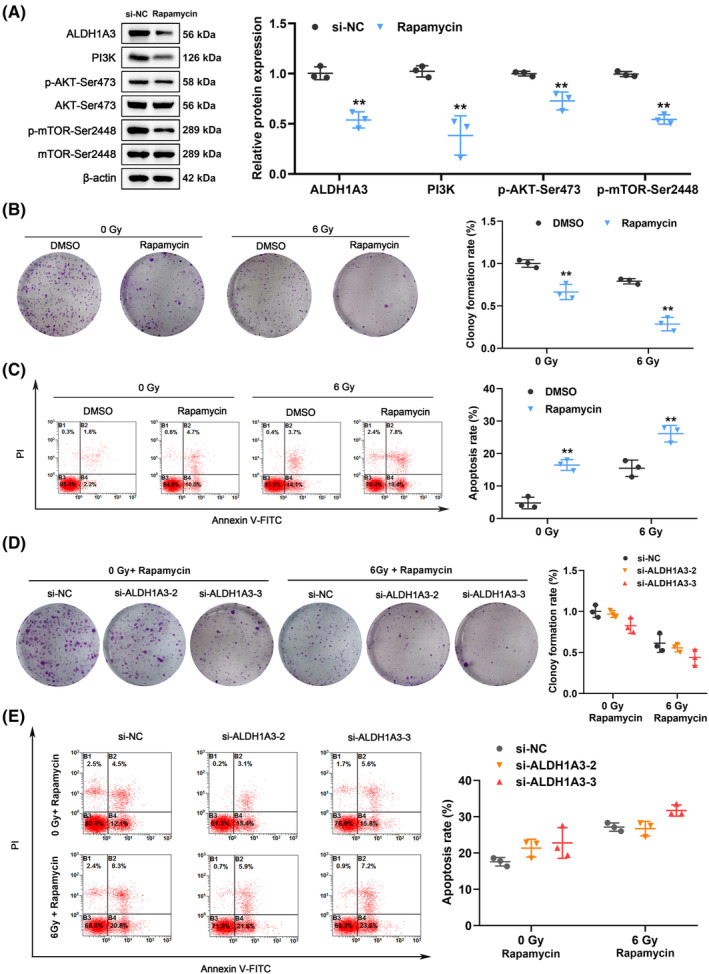
Inhibition of mTOR enhanced radiosensitivity and counteracted the effects of ALDH1A3 on radioresistance in U251 cells. (A) Treatment with 10 nM rapamycin, an mTOR inhibitor, significantly suppressed the expression of ALDH1A3 and PI3K/AKT/mTOR in U251 cells, compared with DMSO treatment. (B) After 24 h of treatment with 10 nM rapamycin, a significant decrease in colony‐forming potential was observed in U251 cells, when exposed to various radiation doses. (C) Combination of 10 nM rapamycin and 6 Gy radiation resulted in a higher rate of apoptosis in U251 cells. (D) Promotion of radiosensitivity through ALDH1A3 silencing was compromised in U251 cells, following pretreatment with 10 nM of rapamycin. (E) Rapamycin counteracted the impact of inhibiting ALDH1A3 on U251 cells apoptosis rate, after 6 Gy radiation. NC represents the negative control. **p < 0.01.

To further investigate the potential role of ALDH1A3 and the PI3K/AKT/mTOR signaling pathway in radioresistance, U251 cells were treated with rapamycin, an mTOR inhibitor. In comparison with DMSO, the use of 10 nM rapamycin significantly decreased the expression of ALDH1A3 and PI3K/AKT/mTOR (Figure 5A). As shown in Figure 5B, U251 cells colony formation was significantly reduced after treatment with 10 nM rapamycin for 24 h, compared with the control group, at various radiation doses. Furthermore, the rate of apoptosis in U251 cells significantly increased when treated with 10 nM of rapamycin and exposed to varying levels of radiation (Figure 5C). Our previous data have revealed that si‐ALDH1A3 enhances radiosensitivity in U251 cells (Figure 1H,I). However, as observed in Figure 5D, rapamycin partially blocked the impact of inhibiting ALDH1A3 on the promotion of radiosensitivity. Moreover, the impact of inhibiting ALDH1A3 on the apoptosis rate of U251 cells was abrogated by rapamycin at varying radiation doses (Figure 5E). Overall, we demonstrated that inhibition of mTOR increased sensitivity to radiation and counteracted the effects of ALDH1A3 on radioresistance in GBM cells.

### 3.6 ALDH1A3 silencing or miR‐4524b‐5p overexpression suppressed in vivo tumorigenesis and radioresistance of GBM cells

**FIGURE 6:**
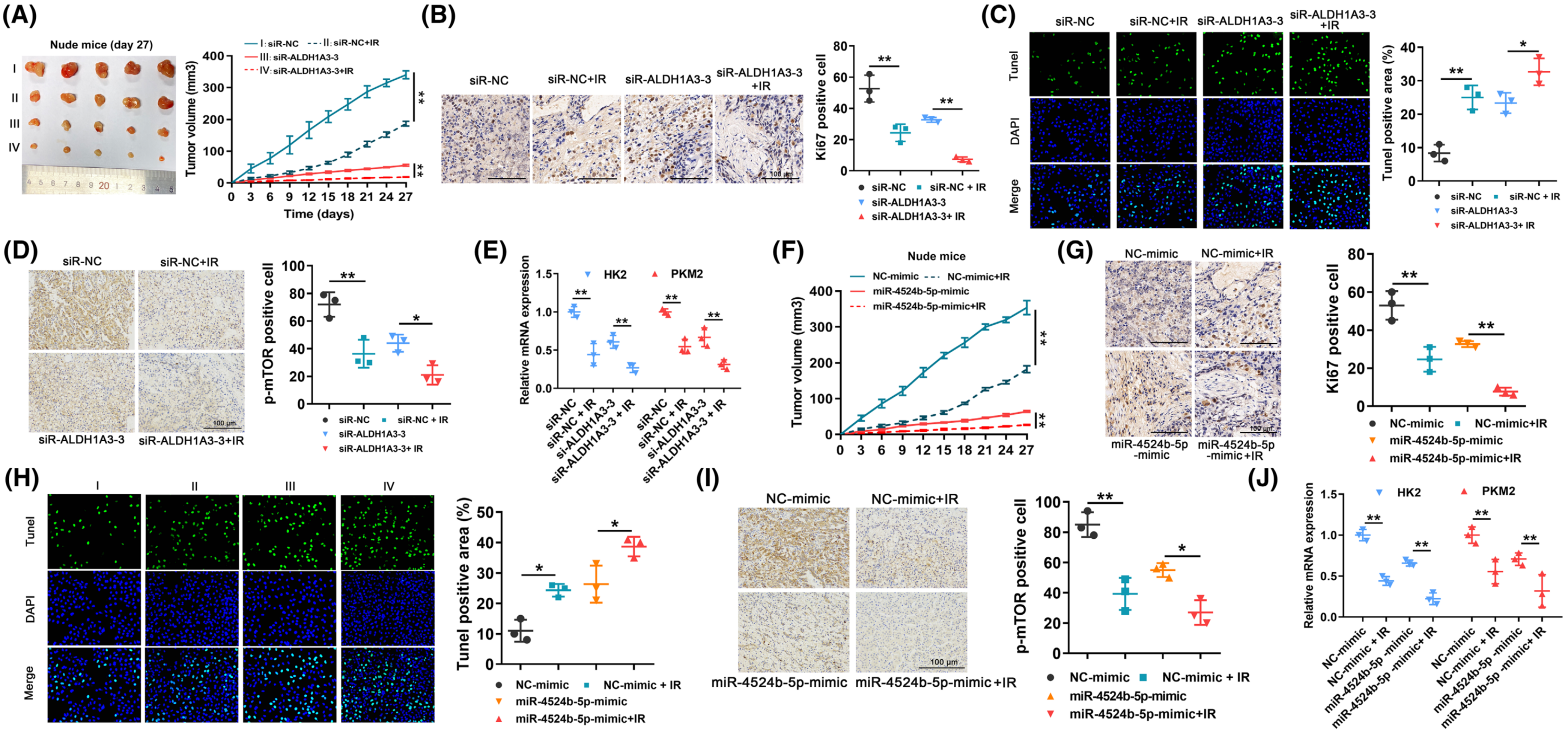
ALDH1A3 silencing or miR‐4524b‐5p overexpression suppressed in vivo tumorigenesis and radioresistance of GBM cells. (A) Xenograft nude mice that were either exposed to 6 Gy radiation or had si‐ALDH1A3 GBM cells implanted, experienced a significant decrease in tumor size, compared with the control group. (B) Ki‐67 expression was suppressed in xenograft nude mice that received either si‐ALDH1A3 U251 cells or 6 Gy radiation, compared with the control group. (C) According to the TUNEL assay, transfection of si‐ALDH1A3 or exposure to 6 Gy radiation increased levels of cell death within the cancerous region of nude mice. (D) Expression of p‐mTOR decreased in the subcutaneous tumors of nude mice that were implanted with si‐ALDH1A3 U251 cells or exposed to 6 Gy radiation. (E) Decrease HK2 and PKM2 levels were observed in the subcutaneous tumors of nude mice that were implanted with si‐ALDH1A3 U251 cells or exposed to 6 Gy radiation. (F) U251 cells tumorigenesis was reduced by miR‐4524b‐5p mimic or 6 Gy radiation. (G) Decrease Ki‐67 expression was observed in xenograft nude mice that had either received 6 Gy radiation or were implanted with miR‐4524b‐5p mimic U251 cells. (H) Overexpression of miR‐4524b‐5p and exposure to 6 Gy radiation increased cell death in the tumor region of nude mice. (I) P‐mTOR amount decreased in the subcutaneous tumors of nude mice, after exposure to 6 Gy radiation or following implantation of miR‐4524b‐5p mimic U251 cells. (J) Overexpression of miR‐4524b‐5p and exposure to 6 Gy radiation decreased mRNA expression of HK2 and PKM2 in xenograft nude mice. NC represents the negative control. *p < 0.05, **p < 0.01.

A series of in vivo experiments was conducted to evaluate the impact of silencing ALDH1A3 or overexpressing miR‐4524b‐5p on tumorigenesis and radioresistance in GBM. The tumor volume of the xenograft mice was measured every 3 days, using Vernier calipers. The group that received a radiation dose of 6 Gy showed a significant decrease in tumor volume, compared with the control group (Figure 6A). Furthermore, immunocompromised mice implanted with si‐ALDH1A3 U251 cells had smaller subcutaneous tumors than mice that received si‐NC cells, when exposed to 6 Gy radiation (Figure 6A). Tumor growth was reduced in immunocompromised mice that were implanted with si‐ALDH1A3 U251 cells and subjected to radiation therapy, as evidenced by a decrease in the levels of Ki‐67 expression (Figure 6B). The TUNEL assay indicated that transfection with si‐ALDH1A3 or exposure to radiation resulted in a higher level of cell death in the tumor area of immunocompromised mice (Figure 6C). Moreover, we observed a reduction in the expression of p‐mTOR, HK2, and PKM2 in the subcutaneous tumors of immunocompromised mice that were implanted with si‐ALDH1A3 U251 cells or exposed to radiation (Figure 6D,E). These results demonstrated that silencing ALDH1A3 inhibited in vivo tumorigenesis and radioresistance of U251 cells. As shown in Figure 6F, transfection with miR‐4524b‐5p mimic or radiation treatment reduced in vivo U251 proliferation. We also observed a decrease in Ki‐67 expression levels, thus indicating a tumor growth reduction in nude mice that received radiation treatment, after being implanted with miR‐4524b‐5p mimic U251 cells (Figure 6G). TUNEL assay results showed that overexpression of miR‐4524b‐5p and exposure to radiation significantly increased cell death within the tumor region of nude mice (Figure 6H). Furthermore, we showed a decrease in p‐mTOR, HK2, and PKM2 levels in the subcutaneous tumors of nude mice that were either treated with miR‐4524b‐5p mimic U251 cells or exposed to radiation (Figure 6I,J). Therefore, results revealed that overexpression of miR‐4524b‐5p reduced in vivo tumorigenesis and radioresistance of U251 cells.

## 4 DISCUSSION

Despite the use of radiotherapy and chemotherapy, following maximum tumor resection, the prognosis of GBM patients remains poor with 5‐year survival rates of 5%.^24^ It has been revealed that radioresistance is a major obstacle that limits the effective treatment of GBM.^25^ Increasing evidence has demonstrated that aberrant expression of miRNAs contributes to tumorigenesis, cancer progression, and therapy resistance in distinct types of cancer.^26^, ^27^, ^28^ A recent study has shown that increased levels of miR155HG act as a sponge for miR‐185, thereby promoting annexin A2 expression, which in turn, contributes to GBM growth and progression.^29^ Moreover, miR‐4524b‐5p targets the WTX/β‐catenin pathway to regulate cervical cancer migration and invasion.^30^ In the present study, miR‐4524b‐5p was identified as the upstream regulator of ALDH1A3 signaling, which affects tumor proliferation and radioresistance in GBM. Overexpression of miR‐4524b‐5p resulted in a reduction in ALDH1A3 expression in GBM cells and was associated with poor prognosis in GBM patients. These results suggest that miR‐4524b‐5p acts as a tumor suppressor in GBM.

Growing evidence has shown that ALDH1A3 participates in various biological processes, including development, progression, and therapy resistance in multiple types of cancer.^8^, ^31^ We have previously demonstrated that ALDH1A3 serves as a functional marker for mesenchymal glioma stem cells, which are characterized by radioresistance and activated glycolytic metabolism.^4^ Furthermore, a recent study has shown that ALDH1A3 plays a key role in the lipid peroxidation of GBM cells, leading to resistance to temozolomide therapy.^32^ Our data suggest that ALDH1A3, which is highly expressed in GBM, is strongly associated with poor prognosis and radioresistance in GBM, which agrees with other studies. In addition, silencing ALDH1A3 not only suppressed glycolytic activity but also targeted the PI3K/AKT/mTOR signaling pathway in GBM.

Radiotherapy primarily kills tumor cells by inducing free radical damage to DNA.^33^ It is well known that the PI3K/AKT/mTOR pathway is one of the most critical mechanisms of radioresistance. Its activation may enhance DNA repair potential in cancer cells.^23^, ^34^ Our data showed that silencing ALDH1A3 significantly impaired the activities in the PI3K/AKT/mTOR signaling pathways. In addition, we found that inhibiting mTOR counteracted the effects of ALDH1A3 knockdown on GBM cell proliferation and radioresistance. It has been reported that ALDH1A3 activates the PI3K/AKT/mTOR signaling pathway by cross‐talking with the transcription factor PPARγ in pancreatic cancer.^35^ Furthermore, abnormal expression of ALDH1A3 promotes chemotherapy resistance in prostate cancer, via the PI3K/AKT/mTOR axis.^36^ Our study revealed a potential regulatory mechanism between ALDH1A3 and the PI3K/AKT/mTOR axis, which is consistent with previous studies. Reports have shown that malignant tumor cells utilize aerobic glycolysis to accumulate pyruvate, lactate, and glutathione, thus enhancing their antioxidant activity and resistance to the harmful effects of free radicals and oxidative stress resulting from radiotherapy.^37^ In addition, the metabolic by‐products resulting from aerobic glycolysis pathways form a network that facilitates the scavenging of free radicals and reactive oxygen species (ROS) within tumor cells. This may reduce the effectiveness of radiotherapy treatment.^38^ In this study, we observed a significant decrease in glucose consumption, and lactate and ATP production in GBM cells, following ALDH1A3 suppression. As expected, ALDH1A3 knockdown significantly decreased mRNA and HK2 and PKM2 protein levels, which are key enzymes involved in glycolysis. Taken together, we demonstrated that suppression of ALDH1A3 impaired the efficacy of radiotherapy in GBM by deactivating the PI3K/AKT/mTOR signaling pathway and glycolysis.

In conclusion, the results of the present study indicated that miR‐4524b‐5p directly targets ALDH1A3, which reduces GBM cells proliferation and radioresistance by regulating the PI3K/AKT/mTOR signaling pathways and glycolysis. Therefore, targeting the miR‐4524b‐5p‐ALDH1A3 axis may be a promising novel approach for GBM treatment.

## AUTHOR CONTRIBUTIONS

Conception and design: Ping Mao and Maode Wang; Development of methodology: Ping Mao and Hai Yu; Acquisition of data: Xiaodong Li, Hai Yu, Yi Li, and Tuo Wang; Analysis and interpretation of data: Ping Mao, Xiaodong Li, and Hai Yu; Writing of the manuscript: Ping Mao and Xiaodong Li.

## FUNDING INFORMATION

This study was supported by the National Natural Science Foundation of China (grant nos. 82072781 and 81602207), the Medical Foundation‐Clinical Integration Program of Xi'an Jiaotong University (grant no. YXJLRH2022040), and the Natural Science Foundation of Shaanxi, China (program no. 2021JM‐264).

## CONFLICT OF INTEREST STATEMENT

The authors declare no conflict of interest.

## Supporting information


Data S1.

## Data Availability

The data sets are available from the corresponding author on reasonable requests.
