# Supplementary material for: MiR‐4524b‐5p‐targeting ALDH1A3 attenuates the proliferation and radioresistance of glioblastoma via PI3K/AKT/mTOR signaling
Source: CNS Neurosci Ther. 2023 Aug 8;30(2):e14396. doi: 10.1111/cns.14396 (PMC10848107; doi:10.1111/cns.14396)

The following figures are "Full unedited gel/blot for all the WB Figures " in the manuscript.

Figure 1D ALDH1A3

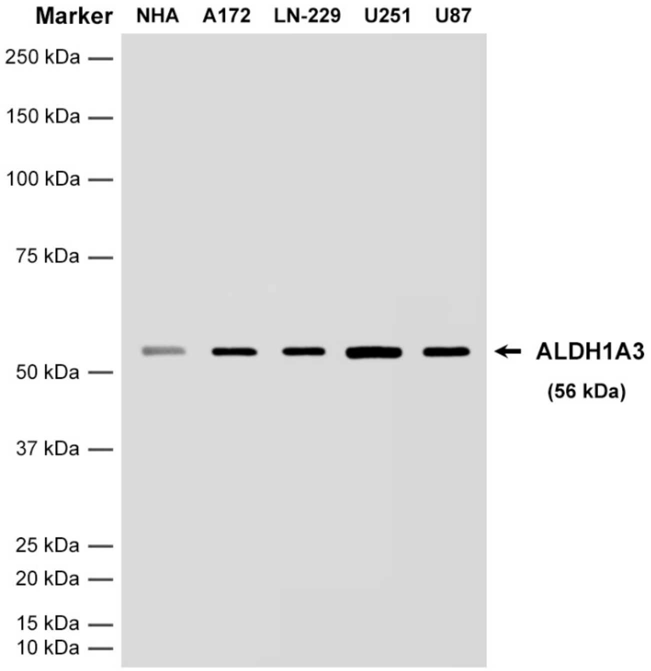

Figure 1D β-actin

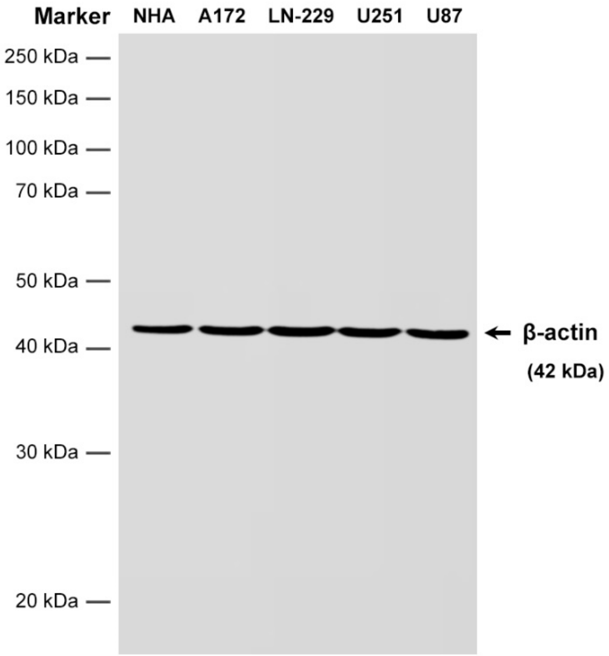

Figure 2B ALDH1A3

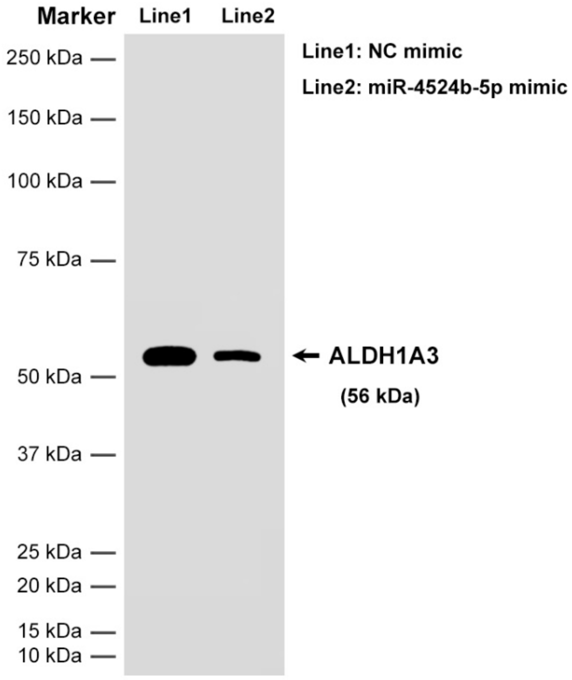

Figure 2B β-actin

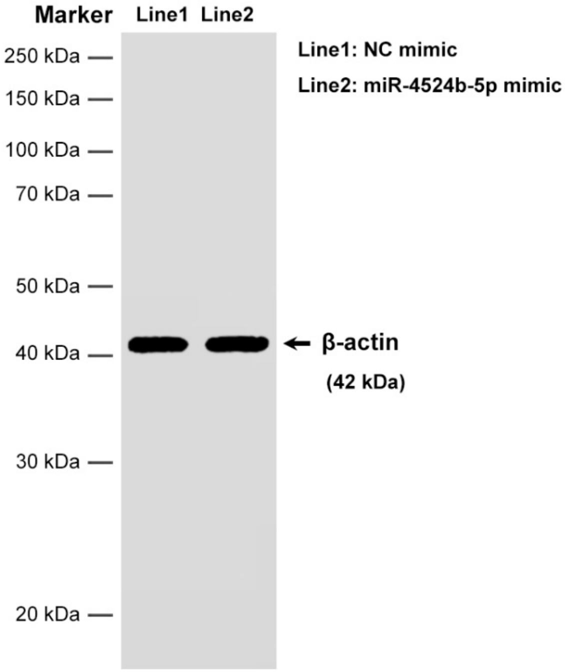

Figure 2C   ALDH1A3

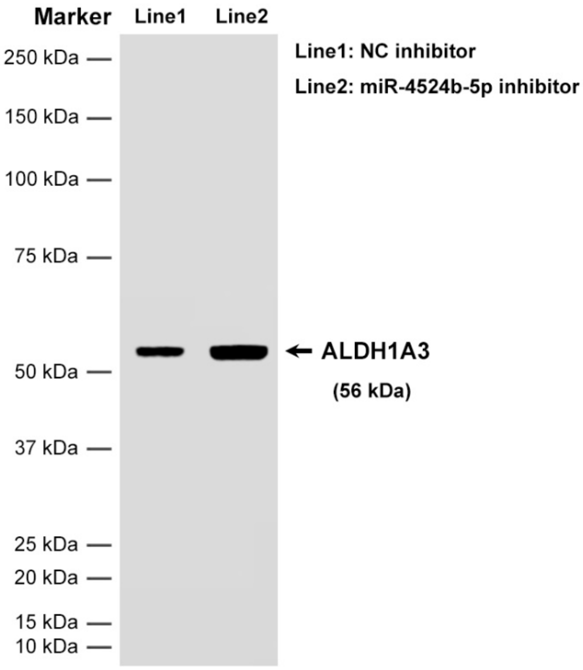

Figure 2C   β-actin

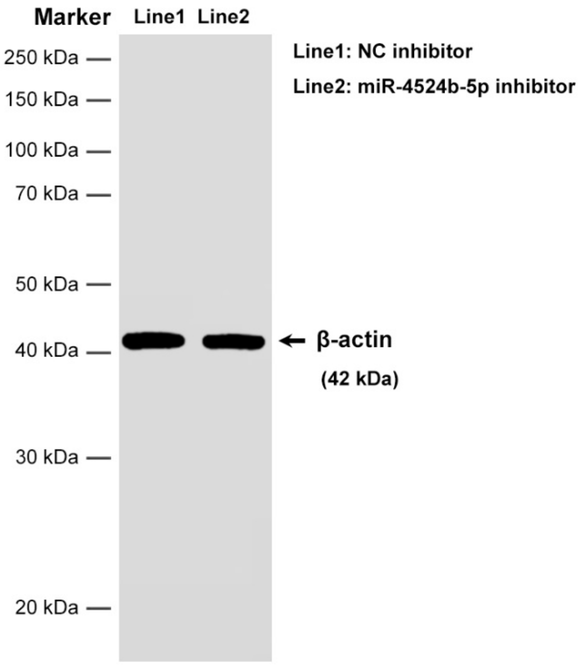

Figure 4A   ALDH1A3

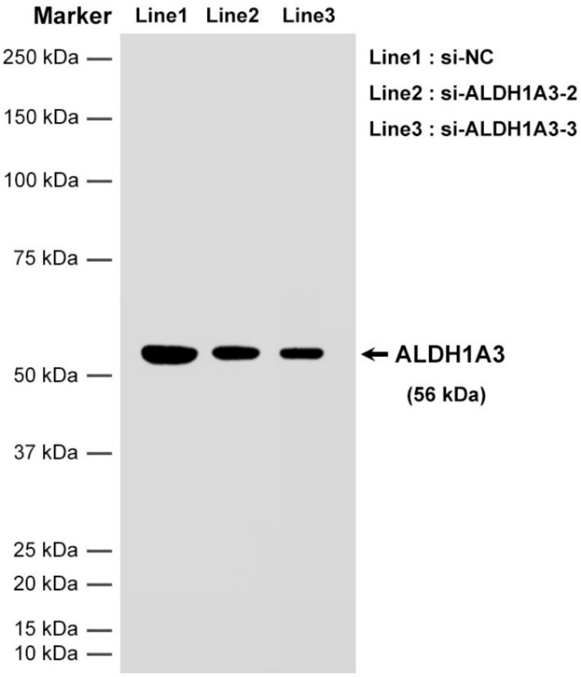

Figure 4A   PI3K

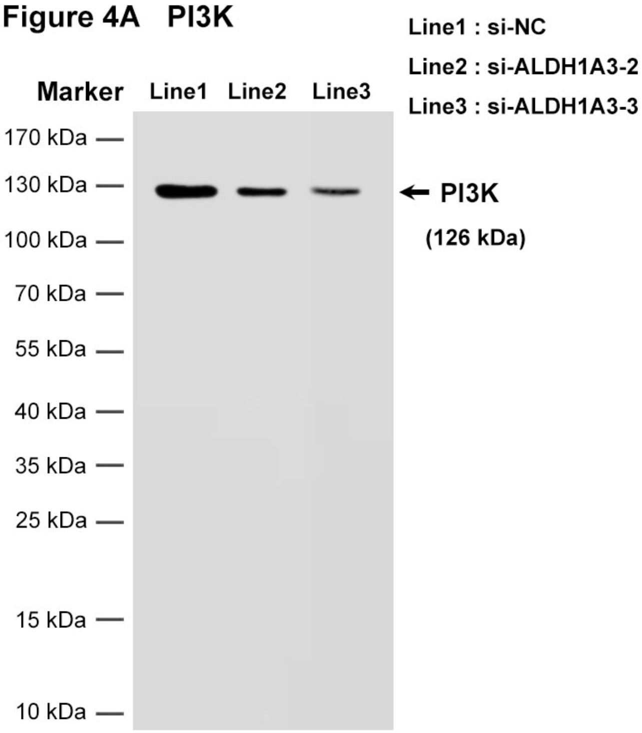

Figure 4A pAKT-Ser473

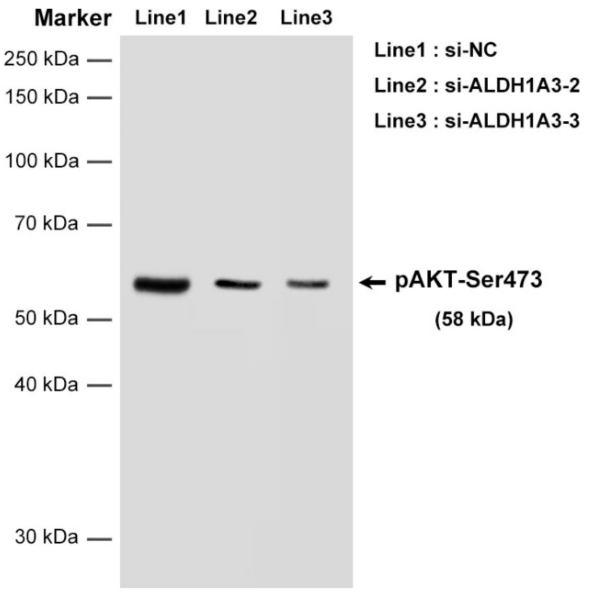

Figure 4A AKT-Ser473

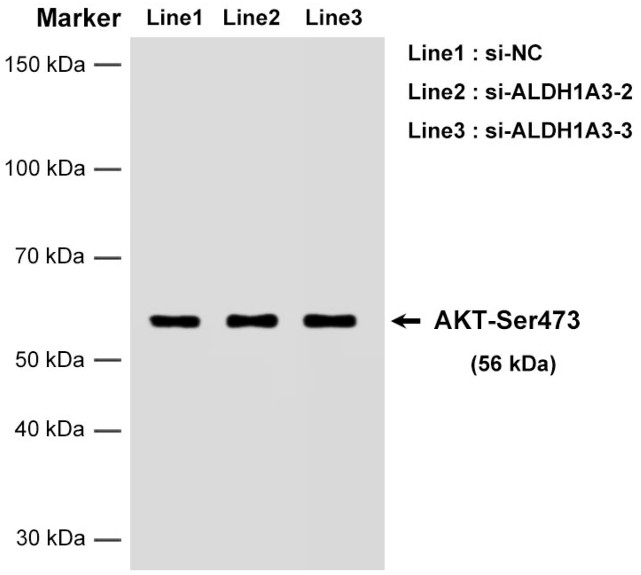

Figure 4A p-mTOR-Ser2448

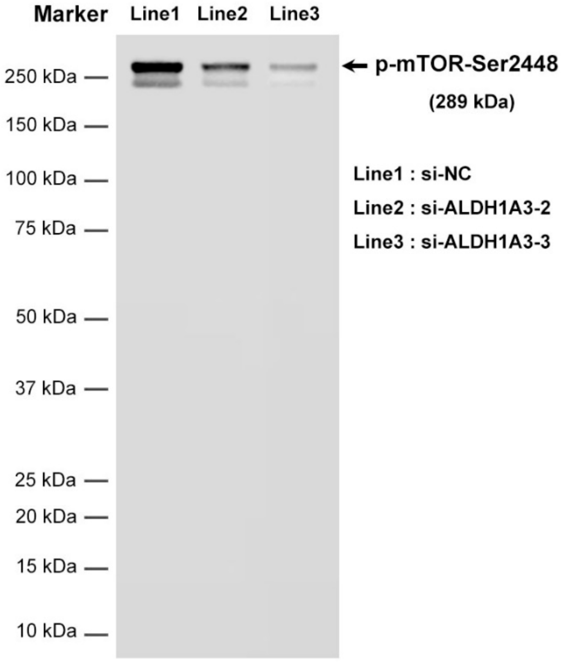

Figure 4A mTOR-Ser2448

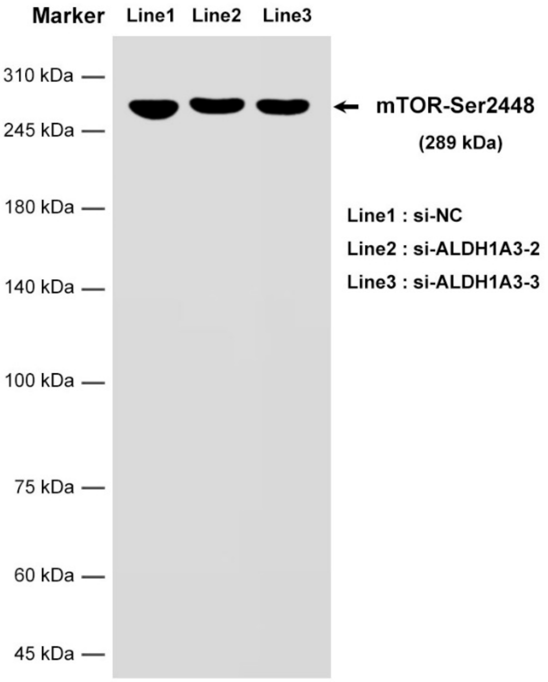

Figure 4A β-actin

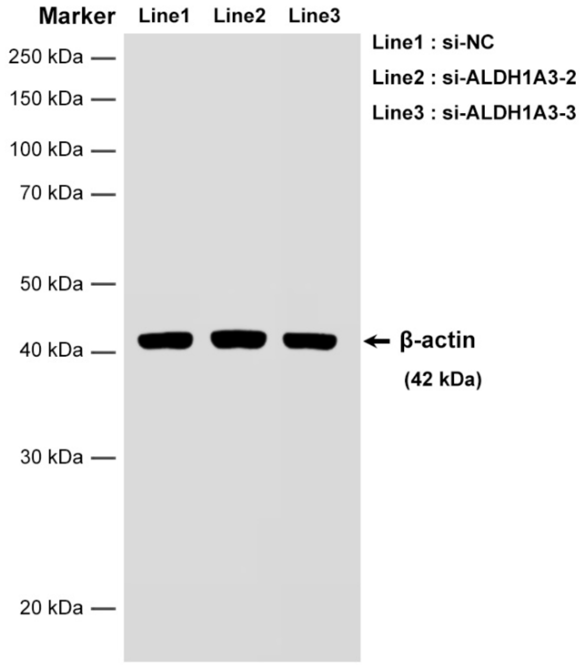

Figure 4D HK2

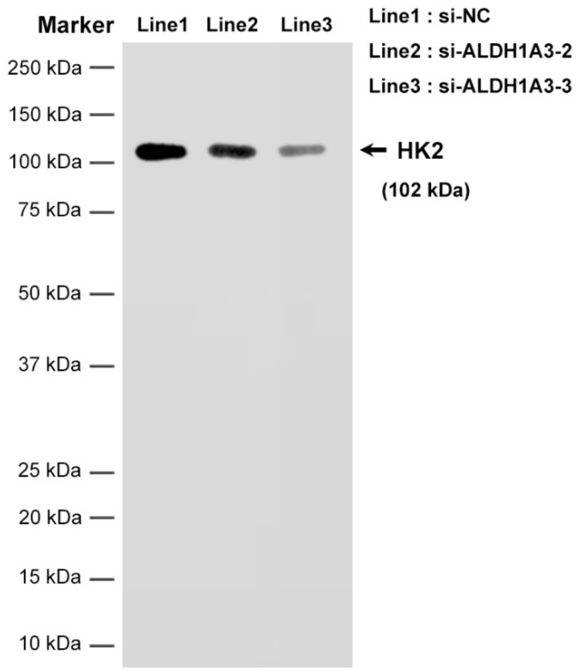

Figure 4D PKM2

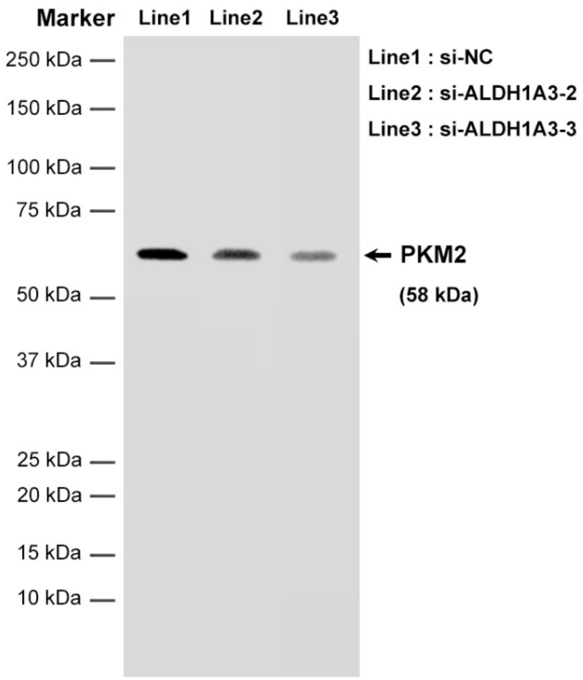

Figure 4D β-actin

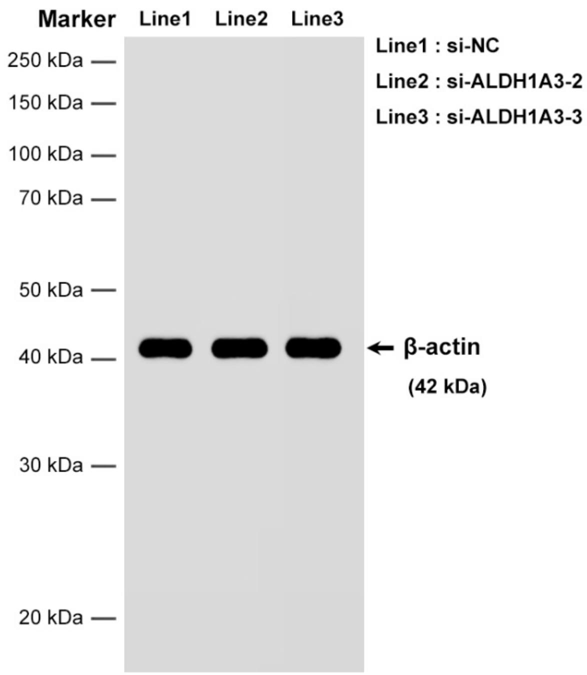

Figure 5A ALDH1A3

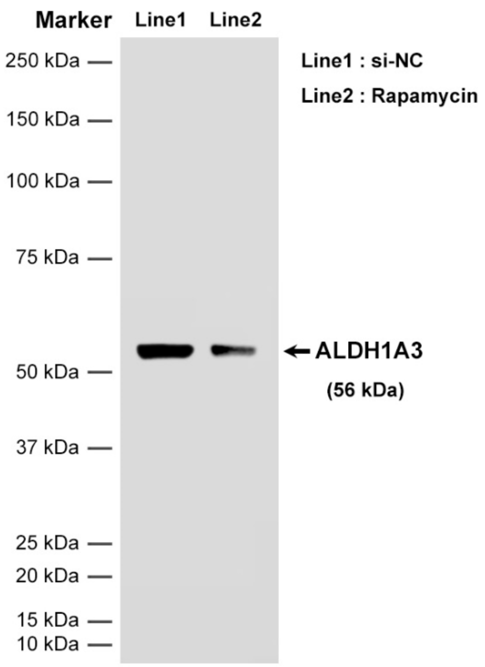

Figure 5A PI3K

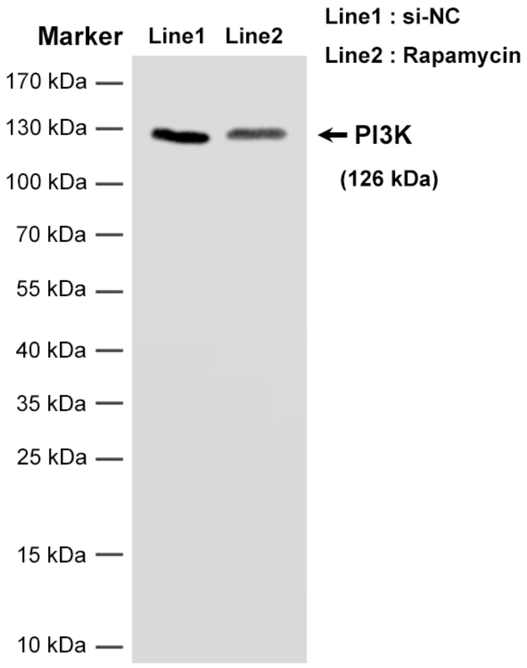

Figure 5A pAKT-Ser473

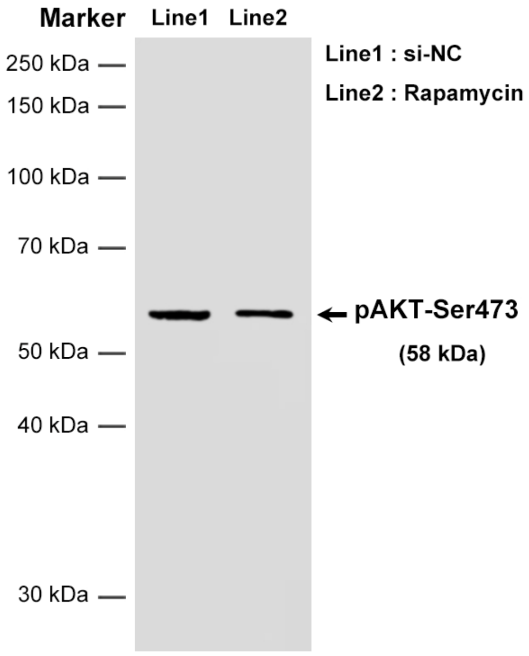

Figure 5A AKT-Ser473

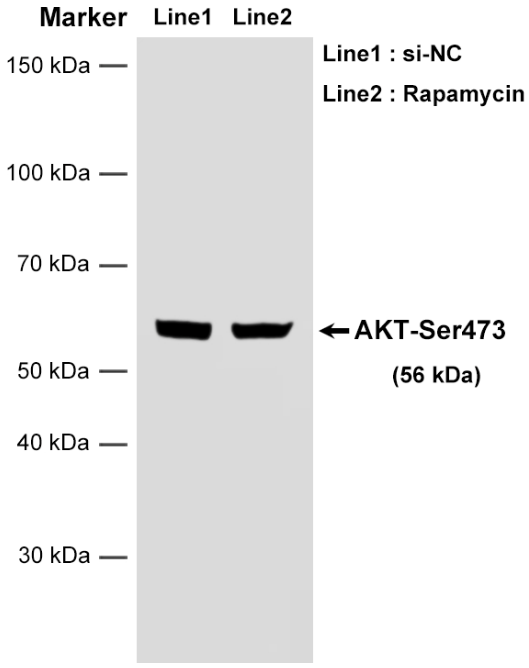

Figure 5A p-mTOR-Ser2448

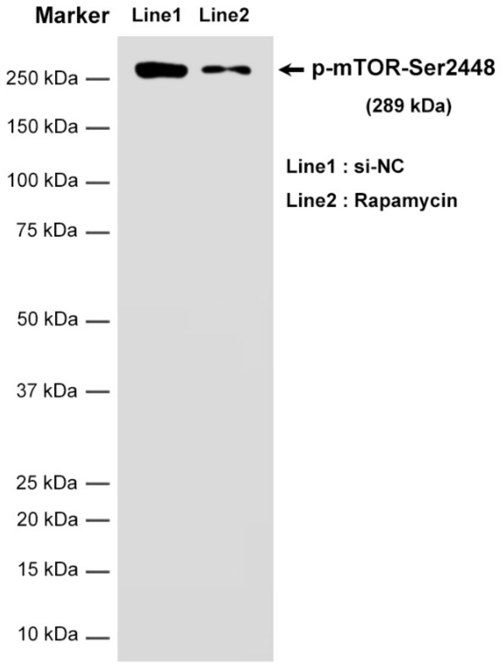

Figure 5A mTOR-Ser2448

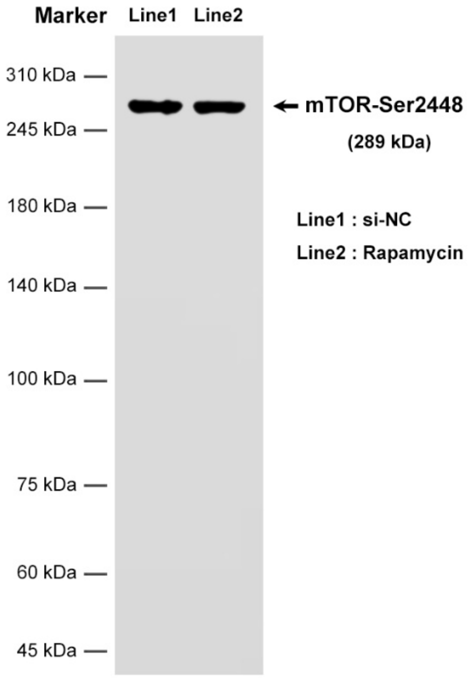

Figure 5A β-actin

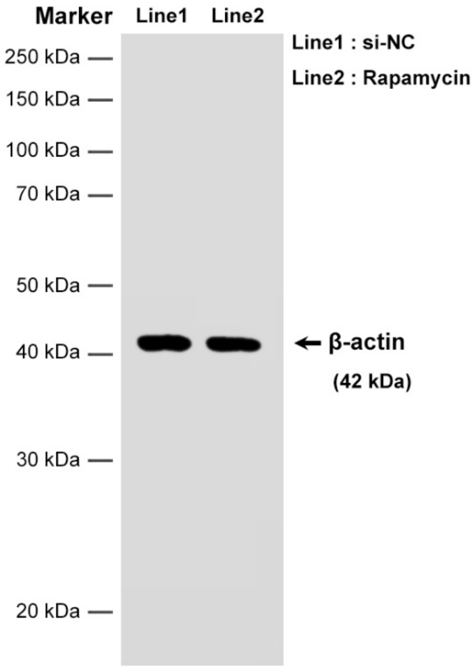

Supplement: Supplementary file 1 — Data S1. [file CNS-30-e14396-s001.pdf]
